# mTOR Pathway is Involved in Energy Homeostasis Regulation as a Part of the Gut–Brain Axis

**DOI:** 10.3390/ijms21165715

**Published:** 2020-08-10

**Authors:** Veronica Pena-Leon, Raquel Perez-Lois, Luisa Maria Seoane

**Affiliations:** 1 Grupo Fisiopatología Endocrina, Instituto de Investigación Sanitaria de Santiago de Compostela, Complexo Hospitalario Universitario de Santiago (CHUS/SERGAS), Instituto de Investigación Sanitaria, Santiago de Compostela, Travesía da Choupana s/n, 15706 Santiago de Compostela, Spain; veritoloren@hotmail.com (V.P.-L.); raquelpl.93@gmail.com (R.P.-L.); 2 Centro de Investigacion Biomedica en Red Fisiopatología de la Obesidad y Nutrición (CIBERobn), 15706 Santiago de Compostela, Spain

**Keywords:** mTOR, gastrointestinal tract, homeostasis, nesfatin-1, ghrelin, obesity

## Abstract

Mammalian, or mechanic, target of rapamycin (mTOR) signaling is a crucial factor in the regulation of the energy balance that functions as an energy sensor in the body. The present review explores how the mTOR/S6k intracellular pathway is involved in modulating the production of different signals such as ghrelin and nesfatin-1 in the gastrointestinal tract to regulate food intake and body weight. The role of gastric mTOR signaling in different physiological processes was studied in depth through different genetic models that allow the modulation of mTOR signaling in the stomach and specifically in gastric X/A type cells. It has been described that mTOR signaling in X/A-like gastric cells has a relevant role in the regulation of glucose and lipid homeostasis due to its interaction with different organs such as liver and adipose tissue. These findings highlight possible therapeutic strategies, with the gut–brain axis being one of the most promising targets in the treatment of obesity.

## 1. Introduction

In 1964, rapamycin was identified, a natural compound with inhibitory action on cell growth and proliferation [1], but the main target of rapamycin action, named mammalian (or mechanic) target of rapamycin (mTOR, also called RAFT1 or FRAP) was not purified in mammals until 1994 [2,3,4].

mTOR is an evolutionarily conserved serine-threonine kinase belonging to the *PI3K-related kinase (PIKK)* family. Regarding its structure, mTOR consists of two different protein complexes, mTOR Complex 1 (mTORC1) and mTOR Complex 2 (mTORC2), that have different functionality, regulation and sensitivity to rapamycin. Both protein complexes share common components such as mTOR, mLST8 (mammalian lethal with sec13 protein 8) and DEPTOR (DEP domain containing mTOR interacting protein) [5,6].

The specific mTORC1 proteins include Raptor (regulatory associated with mTOR) [5] and PRAS40 (proline rich AKT substrate of 40 kda) [7]. On the other hand, the exclusive proteins for mTORC2 are RICTOR (rapamycin sensitive companion of mTOR) [8], Msin1 (stress-activated protein kinase-interacting protein 1) [9] and PROTOR1/2 (protein observed with rictor 1 and 2) [10].

mTORC1 was described as the direct target for rapamycin, while the subunit mTORC2 is insensitive to rapamycin acute treatment [5,11], but longer-term rapamycin treatment suppresses mTORC2 function, likely due to the inability of rapamycin-bound mTOR to incorporate into the new mTORC2 complex [12,13]. When mTORC1 is activated, this inactivates mTORC2 and vice versa. Therefore, mTORC1 and mTORC2 are fundamental pieces of a negative feedback mechanism to maintain proper signaling.

It was described that mTORC1 and mTORC2 have distinct modes of regulation, while the main downstream activated by mTORC1 is p70-S6 kinase 1 (S6K1) and 4EBP1 [11,14]. mTORC2 activation phosphorylates several members of the AGC (PKA/PKG/PKC) family of protein kinases [8,15].

## 2. General Function

mTOR is considered a master regulator of homeostasis, and especially relevant is the regulation of energy balance and peripheral metabolism through its action on the CNS. Among the main general functions of the mTOR signaling pathway, the regulation of cell proliferation, metabolism and protein synthesis have been largely described [16,17].

It was shown that the mTOR signaling pathway has a determinant role in cell growth; specifically, mTORC1 is responsible for a balance between factors that stimulate growth and nutrient availability. Accordingly, mTORC1 is also involved in proliferation mechanisms at the cellular level, since it works as a sensor of nutrient availability in the cell, coordinating the balance between anabolic and catabolic metabolism in response to modifications of exogenous conditions [11,17].

In mammals, changes in energy status are highly dependent on diet, so, under feeding conditions, mTORC1 is activated to promote growth and energy storage in tissues such as the liver and muscles, while, under fasting conditions, mTORC1 is inhibited to conserve limited resources. Metabolic pathways such as glycolysis, the oxidative arm of the pentose phosphate pathway, and de novo lipid biosynthesis have been shown to be stimulated by mTORC1 activation. Moreover, mTORC1 suppresses autophagy [11,17,18].

Although mTORC2 signaling is less understood than mTORC1, recent studies demonstrate that mTORC2 modulates cell metabolism and regulates the organization of the actin cytoskeleton and improves cell survival due to its activation of survival kinase AKT [19]. It has also been implicated in the control of neuron size, neuronal morphology and synaptic plasticity [20] and in the central regulation of energy balance [21].

A wide spectrum of physiological functions has been described for the mTOR complex depending on the tissue in which it is expressed.

### 2.1. Hypothalamus

The hypothalamus is the central organ integrating nutritional and hormonal signals involved in energy balance regulation [22]. In response to changes in energy status, the hypothalamus generates neuronal responses that regulate physiological functions in peripheral organs, such as food intake, glucose homeostasis, thermogenesis and lipid metabolism [16,17,21].

The hypothalamus is constituted by a network of neurons organized in different hypothalamic nuclei. Among them, the arcuate nucleus (ARC), located next to the third ventricle, and the median eminence, have two neuronal populations with opposite effects on energy balance: the agouti-related protein (AgRP)/neuropeptideY (NPY)-producing neurons increase food intake and adiposity, while the activity of pro-opiomelanocortin (POMC)/cocaine and amphetamine-regulated transcript (CART)-producing neurons decrease food intake and adiposity. These neurons interact with other areas involved in the regulation of energy balance, such as the paraventricular nucleus (PVN), dorsomedial nucleus (DMH), ventromedial nucleus (VMH) and lateral hypothalamic area (LHA) [23,24].

At the central level, mTOR regulates several hypothalamic circuits involved in energy balance regulation in response to peripheral signals related to energy status. Between the main hypothalamic nuclei expressing the phosphorylated active forms of mTOR and S6K, in the arcuate nucleus, mTOR co-localizes with key neuropeptides involved in appetite regulation, such as NPY and AgRP [25,26] and POMC clusters [27]. In fact, at the hypothalamic level, the mTOR pathway is regulated by nutritional status, responding to the availability of nutrients and the hormonal environment that modulates the neuronal circuits of the CNS to regulate metabolism and energy balance. Actions of the mTOR central pathway include control of food intake and body weight [16,25,28].

mTORC1 acts as a sensor of energy status in the hypothalamus [25,29,30]. The activity of mTORC1 in the hypothalamus is complex and can change by cell and stimulus type. Hypothalamic signaling of mTORC1 is involved in determining the appetite-reducing effects of nutrients, such as leucine and glucose, and hormones, such as leptin, ghrelin and triiodothyronine [16,31].

Notably, nutrient-activated hypothalamic mTORC1 signaling is specific to neurons or areas. Importantly, although hypothalamic mTORC1 signaling is a critical mediator of the actions of hormones and nutrients in energy metabolism throughout the entire body, it remains to be investigated how mTORC1 signaling in different neuron populations is regulated by hormonal factors and nutrients, as it regulates distinct neuronal activity and function. Moreover, it is still unclear whether mTORC1 interacts with other energy sensors in the hypothalamus to monitor energy balance [31,32].

Nevertheless, it was recently proposed that hypothalamic interplay between the mTOR pathway and the 5′-adenosine monophosphate-activated protein kinase (AMPK) pathway exists to regulate energy balance. This relationship was initially proposed based on the fact that AMPK is able to regulate the activity of S6K and 4EBP1 [33,34], both of which are downstream targets of mTOR. Supporting the relationship between both pathways, the AMPK pathway functions as an energy sensor in the organism and its hypothalamic activity is highly regulated by nutritional status. The regulation of AMPK by nutritional status contrasts that described for mTOR; therefore, in fasting states, AMPK activity is increased in different hypothalamic regions, while refeeding inhibits its activity [35]. This means that the opposite regulation by nutritional status occurs at the hypothalamic level for AMPK and mTOR. Different in vitro and in vivo studies have shown that activation of AMPK suppresses mTOR signaling [32,36].

On the other hand, mTORC2 seems to be involved in the regulation of neuronal morphology and synaptic activity. However, its function in the central regulation of the energy balance is less well understood [20].

### 2.2. Liver

The liver is a critical organ for systemic metabolism. In fasting state, the liver provides energy sources to peripheral tissues through ketogenesis. It was described that, in fasting states, the inhibition of mTORC1 is essential for the correct production of ketonic bodies and for maintaining glucose levels during starvation. It is also fundamental to induce the autophagy in the liver needed to provide amino acids for gluconeogenesis [37]. Regarding glucose homeostasis, the involvement of mTOR was also reported in the control of pancreatic β-cell function [38]. Its role in regulating insulin sensitivity was reported since the overstimulation of mTOR pathway promotes insulin resistance [39].

Hepatic mTORC2 regulates glucose and lipid metabolism by AKT signaling. Additionally, mTORC2 regulates gluconeogenesis and lipogenesis through various transcription factors, including FOXO1, FOXA2 and PPAR-γ [40]. In addition, it was also shown that mTORC1 activation induces de novo lipogenesis in hepatocytes [18,41]. mTORC1 regulates hepatic lipid metabolism mainly through SREBP1, the master regulator of lipid synthesis [42].

### 2.3. Adipose Tissue

The main adipose tissues are white adipose tissue, which stores energy in the form of drops of triglycerides, and brown adipose tissue, which dissipates energy through decoupled breathing and heat production. mTOR has been shown to positively regulate adipogenesis and lipogenesis while inhibiting lipolysis, fatty acid oxidation and ketogenesis.

The molecular mechanisms by which mTORC1 controls adipogenesis involve C/EBP-α and PPAR-γ, among other molecular targets [43]. mTORC2 also plays a critical role in modulating lipid synthesis [44]. Furthermore, the conversion between white and brown adipose tissue also requires the action of mTOR signaling, with a fundamental role in the whole body thermogenesis program [45,46].

## 3. Gut–Brain Axis

The gut–brain axis has been claimed as a key regulator of food intake and energy metabolism. New signals secreted by endocrine cells throughout the gastrointestinal tract are continuously discovered; they affect the hypothalamic centers responsible for regulating energy metabolism through its action not only on food intake but also through the modulation of different processes, such as thermogenesis and lipid metabolism, with the final effect of regulating body weight [47,48]. The mTOR/S6k intracellular pathway has been shown to be involved in the modulation of the production of different signals such as ghrelin and nesfatin-1 in the gastrointestinal tract to regulate food intake and body weight.

### 3.1. The mTOR Pathway in the Gastrointestinal Tract Regulates the Production of Gastrokines Involved in Energy Homeostasis Regulation

In the hypothalamus, the mTOR pathway is proposed as an energy sensor that monitors nutrient availability and regulates energy balance and food intake [25]. In addition to its effects on the hypothalamus, recent research has revealed that mTOR is also involved in the control of energy homeostasis in peripheral organs and especially in the gastrointestinal tract. This is based on the expression of the main components of this intracellular pathway in gastric cells and its effects on the regulation of key gastrointestinal hormones [25,47,49,50,51].

At the gastric level, the expression of the main components of the mTOR pathway is regulated by changes in nutritional status, with low levels of activation in fasting conditions returning to basal expression after refeeding [49]. These findings are supported by several in vitro studies showing that mTOR signaling is activated in the presence of increased intracellular ATP levels [52]. In pathological states, increased activity of the mTOR signaling has been associated with obesity [39] and, accordingly, the knockout mice for the mTOR downstream S6kinase 1 are protected against diet-induced obesity [53].

It was reported that the components of the mTOR pathway are expressed in different endocrine cells, including X/A-like cells and with lower expression in G cells [54]. Specifically, in X/A-like cells, one of the most abundant types of gastric endocrine cells, the mTOR pathway is involved in the secretion of different gastrokines with a relevant role in metabolism regulation such as ghrelin and nesfatin-1 [53]. Consequently, this pathway has been proposed as a component of the gut–brain axis by modulating the gastric production of different stomach-derived signals involved in energy homeostasis [49,50,51].

#### 3.1.1. mTOR and Ghrelin

The role of the gut–brain axis in the regulation of food intake and energy homeostasis became relevant after ghrelin isolation in 1999. Ghrelin is the only described anorexigenic peptide released from the gastrointestinal tract, regulating energy homeostasis by increasing food intake and adiposity [55]. Ghrelin is synthetized in the in X/A-like cells of the gastric fundus and its levels are regulated by nutritional status [56,57]. This fact, together with the emerging role of the mTOR pathway in the gastrointestinal tract as a nutrient sensor, evidences a potential interaction between ghrelin and this intracellular pathway in the control of energy homeostasis in response to nutrient ingestion. It was widely described that, in fasting states, ghrelin secretion by the stomach is increased [58], and this nutritional condition is also related to a down-regulation of the mTOR pathway, which suggests a gastric inverse pattern between ghrelin and mTOR signaling during changes in energy status. In fact, Xu et al. in 2009 described that the inhibition of mTOR signaling in the stomach after rapamycin treatment induces an increase in the production of gastric ghrelin and in the levels of the enzyme responsible for ghrelin acylation, GOAT, resulting in an increase in circulating levels of ghrelin. Accordingly, the activation of mTOR by L-leucine treatment down-regulates gastric ghrelin production and decreases its circulating levels [49].

#### 3.1.2. mTOR and Nucb2/nesfatin-1

The involvement of the mTOR pathway in fuel sensing at the gastric level for the maintenance of energy homeostasis should not only be considered in relation to its effects on ghrelin regulation. Certainly, new gastrokines involved in the gut–brain axis’ regulation of body weight are continuously discovered [47]. One of these hormones is Nucb2/nesfatin-1, which is considered an anorexigenic signal expressed mainly in the gastric mucosa [59], although its expression was also described in a lesser extent in other central [60,61] and peripheral tissues [62]. At the cellular level, it was described as being responsible for expression of Nucb2/nesfatin-1 in X/A-like cells that also contain ghrelin, although it is located in a different pool of cytoplasmic vesicles to those producing ghrelin [59]. The peripheral nesfatin-1 has been shown to be able to cross the blood–brain–barrier to affect food intake at the central level [63]. As described for ghrelin production in the stomach, nesfatin-1 is also regulated at the gastric level by nutritional status, but the pattern of regulation is opposed to those described for ghrelin with lower levels of circulating nesfatin-1 under fasting conditions [64,65]. Based on these findings, the modulation of gastric Nucb2/nesfatin-1 has been proposed as a promising therapy for the treatment of obesity. Interestingly, Nucb2/nesfatin-1 expression in gastric mucosa co-localizes with the mTOR signaling molecule pS6k1 and a matching relationship between gastric nesfatin-1 production and mTOR signaling occurs at the gastric level under alterations in nutritional status and also in pathological processes such as obesity. Together, these findings indicate that gastric Nucb2/nesfatin-1 is regulated by mTOR [66].

#### 3.1.3. The mTOR Pathway Modulates the Production of Gastrokines Acting Partially through the Gastric Cannabinoid System

An interaction between the gastric mTOR pathway and the cannabinoid system was recently described to regulate the production of the main gastrokines involved in energy homeostasis, such as ghrelin and nesfatin-1.

A few years ago, a novel mechanism of food intake regulation was described in the stomach by an interaction between the gastric cannabinoid system and ghrelin, orchestrated by the mTOR signaling pathway [50]. The receptor CB1, a main regulator of appetite control [67], is co-expressed in the endocrine cells of the gastric fundus with ghrelin [68]. The pharmacological blockade of cannabinoid receptor (CB1) by treatment with the inverse agonist rimonabant reduces food intake and increases mTOR/S6k1 activity in the stomach. However, this effect is only observed in fasting animals with high levels of ghrelin, which are reduced after treatment with rimonabant in parallel with the activation of the mTOR/S6k1 pathway. Supporting these data, after the pharmacological blockade of the mTOR pathway by chronic treatment with rapamycin, rimonabant is not able to affect ghrelin secretion. In this context, the modulation of mTOR activity in the stomach is a key mechanism mediating the effect of the cannabinoid system on ghrelin production and ultimately regulating food intake.

Moreover, the effect of this gastric system on central food intake was carried out through neural control of the vagus nerve [50]. The pathway mTOR turns out to be a crucial component of the gut–brain axis, regulating food intake. The main connection between the brain and the periphery is the vagus nerve [58]. Accordingly, it was previously shown that, in fasting states, the production of ghrelin in the stomach is increased and this exerts its orexigenic effects in the brain via the activation of the vagus nerve [58,69]. Supporting this, it was shown that ghrelin is not able to induce food intake in animals after surgical vagotomy and, furthermore, the inhibition of ghrelin induced by rimonabant treatment is also blocked in vagotomized animals. In summary, the gastric mechanism involving the interaction between the cannabinoid system and ghrelin is mediated by the intracellular mTOR pathway and requires an intact vagus nerve in order to produce central effects on the regulation of food intake.

However, the described gastric interaction between the cannabinoid system and mTOR is not only involved in ghrelin production. It was recently reported that the mTOR pathway mediates the interaction between the cannabinoid system and nesfatin-1 to modulate food intake. The inhibition of cannabinoid receptor 1 (CB1) by the peripheral injection of rimonabant decreases food intake and induces an increase in the gastric production of Nucb2/nesfatin-1, along with the activation of the mTOR pathway in the stomach. Confirming these findings, the inactivation of the intracellular pathway mTOR/S6k by chronic treatment with rapamycin blocks the stimulatory effect of rimonabant in the gastric secretion of Nucb2/nesfatin-1 [51].

These findings altogether reveal that the intracellular mTOR pathway in the stomach mediates a gastric mechanism in which the cannabinoid system regulates the production of the anorexigenic hormone Nucb2/nesfatin-1 and the orexigenic ghrelin in an opposing manner. This works as a mechanism to balance anorectic and orexigenic signals to maintain energy homeostasis. The gastrointestinal tract and gastrokines, together with novel systems such as the endocannabinoid system and the intracellular mTOR pathway, represent promising pharmacological targets for the development of therapies against obesity.

## 4. Modulation of mTOR Signaling in Gastric X/A Cells Presents Extra Gastric Effects

The role of gastric mTOR signaling in different physiological processes was deeply studied by using different genetic models that allow the modulation of mTOR signaling specifically in the stomach. The first of these knocked-down TSC1 in animal models, a key component of mTOR signaling in the stomach, whose inhibition induces the activation of mTOR signaling. This is an experimental model of mTOR activation specifically in the stomach, without effects on other organs in which mTOR is expressed. In addition, these mice present decreased ghrelin expression, food intake and lipid content in the liver.

The described knockout model affects the global gastric cells expressing mTOR. However, the most interesting model is the one which selectively targets the mTOR pathway in gastric X/A cells, the endocrine cells that produce the main known gastrokines involved in energy homeostasis regulation, such as ghrelin and nesfatin-1. The mice model of genetic mTOR activation specifically in X/A cells presents decreased body weight and fat adipose content than its wild type without significant differences in food intake, according to unaffected expression of the main hypothalamic genes regulating appetite (NPY, AGRP and POMC). The leaner phenotype of mice with activated mTOR in X/A cells is probably due to a decrease in the gastric expression and secretion of ghrelin [70].

### 4.1. Pancreas Fibrosis

In the hypothalamus, the mTOR pathway is proposed as an energy sensor monitoring nutrient availability. A possible relationship between gastric X/A cells and the fibrotic process in the pancreas leading to insulin resistance has been proposed. The signals such as ghrelin and nesfatin-1 secreted from X/A cells seem to be the nexus between gastric X/A cells and pancreas function. Recent reports have described that the modulation of mTORC1 in gastric X/A cells is critical to maintain the pancreatic microenvironment. Accordingly, it was shown how the activation of the mTORC1 pathway in X/A cells induced the appearance of pancreatic fibrosis in mice, causing glucose intolerance. This effect is mediated by the decreased ghrelin production in X/A cells. In fact, pancreatic fibrosis and insulin intolerance are reversed after replacement of ghrelin by exogenous administration. In the same way, by administering rapamycin, which inhibits mTORC1, it was possible to reverse the decreased ghrelin levels and pancreatic fibrosis [53].

A mouse model with ghrelin production deficiency in X/A cells (Ghrel^−^) and normal production in the pancreas and hypothalamus has no effect on body weight, food intake, plasma ghrelin levels or glucose. When this model (Ghrel^−^) is crossed with mice with activation of gastric mTORC1 (TSC1^−/−^), a new model of mice is generated that presents a decrease in the expression of plasma acyl ghrelin, gastric ghrelin and total ghrelin. In addition, a significant increase in pancreatic fibrosis was found in both thin and obese mice, but pancreatic fibrosis activation was more severe in obese mice. Mice Ghrel-TSC1^−/−^ were found to be resistant to obesity induced by high fat intake and presented lower body weight and food intake.

It is widely known that fibrotic processes in the pancreas result in an increase in the extracellular matrix components in pancreatic tissue, and the main components of the extracellular matrix are degraded by the metalloproteases. In the genetic model of mTORC1 activation in gastric X/A cells, a decreased production of the main metalloproteases MMP9 was detected together with an increase in the inhibitor of metalloproteinase TIMP1. To highlight the importance of decreased ghrelin production in the mice after mTOR activation in X/A, this effect was reverted after administration of ghrelin exogenously to the mice. This treatment reversed the circulating low levels of acyl ghrelin described in the animal model, together with an increase in food intake, which indicates that the activation of mTOR in X/A induces a decrease in food intake that is mediated by the reduction in gastric ghrelin production [53]. This finding is supported by previous articles showing the gastric mechanism regulating appetite and body weight, led by the interaction between ghrelin and mTOR [25,50]. Accordingly, the exogenous administration of ghrelin also improves pancreatic fibrosis, as shown by the decrease in collagen deposition and the increase in pancreatic MMP9 levels. In light of the described mechanism, it would be reasonable to think that the inhibition of mTORC signaling in gastric X/A cells could constitute a therapeutic approach for pancreatic fibrosis in those states with an overactivation of mTOR signaling. In fact, when the main known inhibitor of the mTOR pathway, rapamycin, is injected into genetic mice with gastric mTOR over activation, fibrosis reverts after restoration of circulating ghrelin levels.

### 4.2. Glucose Metabolism

The model of Ghrel-TSC1^−/−^ showed high glucose levels with impaired glucose metabolism, although insulin sensitivity remains unchanged. The authors ruled out the development of insulin resistance in these mice. When insulin secretion and expression was studied in Ghrel-TSC1^−/−^ mice, a decrease in mRNA and protein levels of insulin was found in the pancreas that was associated with a diminution in the levels of insulin in plasma. Furthermore, insulin secretion stimulated by glucose is reduced. This diminution in insulin production was associated with a decrease in pancreatic insulin β-cells. Altogether, this contributes to the altered glucose homeostasis, and decreased levels of insulin in plasma found in the Ghrel-TSC1^−/−^ mice were reverted after ghrelin administration [53]. Any alteration in inflammatory cytokines (TGF β, Smad2 and Smad3) involved in the glucose homeostasis impairment was not found in the genetic mouse model with the activation of mTORC1 in X/A cells. It is interesting that the suppression of ghrelin production in gastric X/A in different genetically engineered mouse models did not affect food intake or body weight; however, it has notorious effects on glucose metabolism under caloric restriction conditions [71].

### 4.3. Hepatic Actions

At the hepatic level, the overexpression of mTOR signaling in X/A cells induces a decrease in lipogenesis and an increase in β-oxidation in the liver, causing a decrease in hepatic lipid content in normal chow diet and high-fat diet mice which is supported by the role of ghrelin in inducing lipogenesis in hepatocytes via a mechanism involving the mTOR signaling pathway [41].

A model of gastric mTORC activation was performed in mice by the knockdown of gastric TSC1. This model presented not only activation of mTOR signaling but also the phosphorylation of the main downstream of mTOR, pS6k. Gastric ghrelin production was significantly reduced in this mouse model, which occurs in parallel with reduced food intake. In addition to the effects described on fat content, a decrease in liver fat was also observed in these mice, which was associated in the literature with decreased ghrelin levels [70]. Consequently, with this lower lipid content, the main hepatic signaling pathways involved in lipogenesis are decreased in the mouse model (srebf1, srebf2, pparγ and pparγ2), and the expression of oxidation related genes is increased.

When mTOR activation in the stomach was selectively performed in gastric X/A-like cells, hepatic lipogenesis was suppressed and β-oxidation was stimulated. Furthermore, it was demonstrated that mTOR activity in X/A type cells regulates hepatic lipid metabolism, partially through ghrelin [70]. This is corroborated by previous studies in which authors observed that ghrelin induces lipogenesis in hepatocytes by mTOR signaling [41].

### 4.4. Adipose Tissue

At the adipose tissue level, mTOR activity in gastric type X/A cells has been reported to contribute to the browning of white adipose tissue. Suppression of mTOR signaling in X/A-like cells is associated with a decrease in browning genes in white adipose tissue. Furthermore, ghrelin may mediate mTOR effectors, as it was noted that circulating ghrelin levels, gastric mTOR activity and brown gene levels have a negative relationship. Interestingly, when exogenous ghrelin is administered, the browning produced by mTOR signaling activation in X/A-like cells is partially reversed. In addition to the effects in the liver, adipose tissue was also affected in the mice with the knockdown of gastric TSC1, as evidenced by the smaller size of adipocytes in parallel with the lower expression of the main genes involved in de novo lipogenesis in adipose tissue and browning (UCP1). Furthermore, these effects are independent of the affected insulin control that these mice showed [70].

## 5. Conclusions

The mTOR/S6k intracellular pathway has been shown to be involved in the gastrointestinal tract in modulating the production of different signals, such as ghrelin and nesfatin-1, to regulate food intake and body weight. Furthermore, with the use of different genetic models that modulate mTOR signaling in the stomach, and specifically in gastric X/A-like cells, the relevant role of this signaling pathway in the regulation of glucose and lipid homeostasis in other tissues such as liver and adipose tissue has become evident.
